# Antimicrobial resistance patterns, virulence gene profiles, and genetic diversity of *Salmonella enterica *serotype Enteritidis isolated from patients with gastroenteritis in various Iranian cities

**DOI:** 10.22038/ijbms.2021.54019.12142

**Published:** 2021-07

**Authors:** Fatemeh Fardsanei, Mohammad Mehdi Soltan Dallal, Taghi Zahraei Salehi, Masoumeh Douraghi, Mojtaba Memariani, Hamed Memariani

**Affiliations:** 1 Division of Microbiology, Department of Pathobiology, School of Public Health, Tehran University of Medical Science, Tehran, Iran; 2 Food Microbiology Research Center, Tehran University of Medical Sciences, Tehran, Iran; 3 Department of Microbiology, Faculty of Veterinary Medicine, University of Tehran, Iran; 4 Skin Research Center, Shahid Beheshti University of Medical Sciences, Tehran, Iran; 5 Department of Bacteriology, Faculty of Medical Sciences, Tarbiat Modares University, Tehran, Iran; 6 Biotechnology Research Center, Pasteur Institute of Iran, Tehran, Iran

**Keywords:** Antibiotic resistance, Gastroenteritis, PFGE, Salmonella enterica serotype – Enteritidis, Virulence genes

## Abstract

**Objective(s):**

This study aimed to evaluate antibiotic resistance profiles and presence of virulence genes among *Salmonella enterica *serovar Enteritidis (*S. *Enteritidis) isolated from patients with gastroenteritis in various regions of Iran. Moreover, genetic relatedness among the strains was assessed by pulsed-field gel electrophoresis (PFGE).

**Materials and Methods:**

From April through September 2017, 59 *Salmonella* strains were isolated from 2116 stool samples. Of these strains, 27 *S. *Enteritidis were recovered. These strains were subjected to disk diffusion tests, polymerase chain reaction (PCR) for detection of virulence genes (*invA, hilA, pefA, rck, stn, ssrA, ssaR, sefA, spvC, sipA, sipC, sopB, sopE*, and *sopE2*), and PFGE.

**Results:**

High prevalence of resistance towards cefuroxime (n = 20, 74.1%) and ciprofloxacin (n = 13, 48.2%) were demonstrated. All tested strains possessed* invA, hilA, sefA, sipA, sopB*, and *sopE*. The least prevalent virulence gene was *rck* (n = 6; 22.2%). Based on combinations of virulence genes, 12 virulotypes were observed. The most common virulotype was VP2 (n = 12; 44.4%), harboring all of the virulence genes except for *rck*. PFGE typing showed only two distinct fingerprints among tested strains. Each fingerprint had completely different virulotypes. Notably, VP4 (harboring all genes except for *rck* and *spvC*) was only presented in pulsotype A, while VP2 was confined to pulsotype B.

**Conclusion:**

*S*. Enteritidis strains were derived from a limited number of clones, suggesting that it is highly homogenous. Future works should consider combinations of other genotyping methods together with larger sample sizes from more diverse sources.

## Introduction

Non-typhoidal *Salmonella* (NTS) serovars are among the most frequently reported causes of gastroenteritis across the globe, in particular in developing countries. It has been estimated that almost 94 million cases of gastroenteritis due to NTS occur annually, culminating in 155,000 deaths per year (1). Thus far, over 2,500 different *Salmonella* serovars have been identified. Nevertheless, *Salmonella enterica* serovar Enteritidis (*S*. Enteritidis) and *S. enterica* serovar Typhimurium still account for close to half of all human isolates of NTS (2). As a food-borne pathogen, *S*. Enteritidis has been associated with a variety of infectious diseases in different animals, including cattle, swine, and poultry. Contaminated animal products such as meat, pork, eggs, and milk are considered to be the main vehicle of transmission to humans (3).

The majority of *Salmonella* gastroenteritis cases are mild, self-limiting, and uncomplicated which seldom require antibiotic therapy. In addition, antibiotics do not diminish the duration of symptoms and may actually lengthen the period of asymptomatic shedding. However, they are reserved for patients with severe or invasive illnesses (4). On the other hand, injudicious and excessive use of antibiotics in livestock and poultry production is somewhat blamed for the emergence of multidrug-resistant (MDR) zoonotic pathogens, which may be transmitted from animals to humans through the food chain (5). Over the course of the past years, the world has witnessed a considerable upsurge in the prevalence of MDR *Salmonella* among patients. As a result, continuous surveillance and monitoring of antimicrobial resistance in foodborne pathogens will provide valuable data to healthcare professionals, researchers, and regulatory agencies (6-8).

Type 3 secretion systems (T3SS) play a prominent role in the pathogenesis of *Salmonella*. These syringe-like machines are specialized for the translocation of virulence determinants, also dubbed effector proteins, directly from the bacterial cytoplasm into host cells (9). In *Salmonella*, two functionally distinct T3SSs, which are encoded by *Salmonella *pathogenicity islands (SPIs), have hitherto been characterized. In this respect, T3SS encoded by SPI-1 delivers effector proteins required for intestinal invasion (10), while SPI-2 is activated intracellularly and is crucial for *Salmonella* replication inside host cells (11).

Over the recent years, there has been a significant rise in the number of salmonellosis cases in Iran. However, the majority of these cases were mostly reported from Tehran (12-16). There is a scarcity of data concerning the prevalence and characterization of clinical *S*. Enteritidis strains from other regions of Iran. Hence, the main objectives of the present study were to evaluate antibiotic resistance profiles and the presence of virulence genes among *S*. Enteritidis isolated from patients with gastroenteritis in various Iranian cities. In addition, we used pulsed-field gel electrophoresis (PFGE) to determine the clonal relatedness and to elucidate any epidemiological links between the *S*. Enteritidis strains.

## Materials and Methods

***Reagents and media***

All of the common chemical reagents used in the current study were of analytical grade from commercial suppliers. Müeller-Hinton broth (MHB), Müeller-Hinton agar (MHA), triple sugar iron (TSI) agar, and tryptic soy broth (TSB) were procured from the Merck Co. (Darmstadt, Germany). Antibiotic disks were purchased from Mast Diagnostics Ltd. (Bootle, United Kingdom). Polyvalent and monospecific antisera against somatic and flagellar antigens were procured from BD Difco^TM^ (Franklin Lakes, NJ, USA). Ultrapure agarose gel powder was supplied from Invitrogen (Carlsbad, CA, USA). Restriction enzyme *Xba*I was obtained from Thermo Fisher Scientific (Waltham, MA, USA). The other materials and reagents were all obtained from Sigma Aldrich Co. (Steinheim, Germany).

***Bacterial isolation***

**Figure 1:**
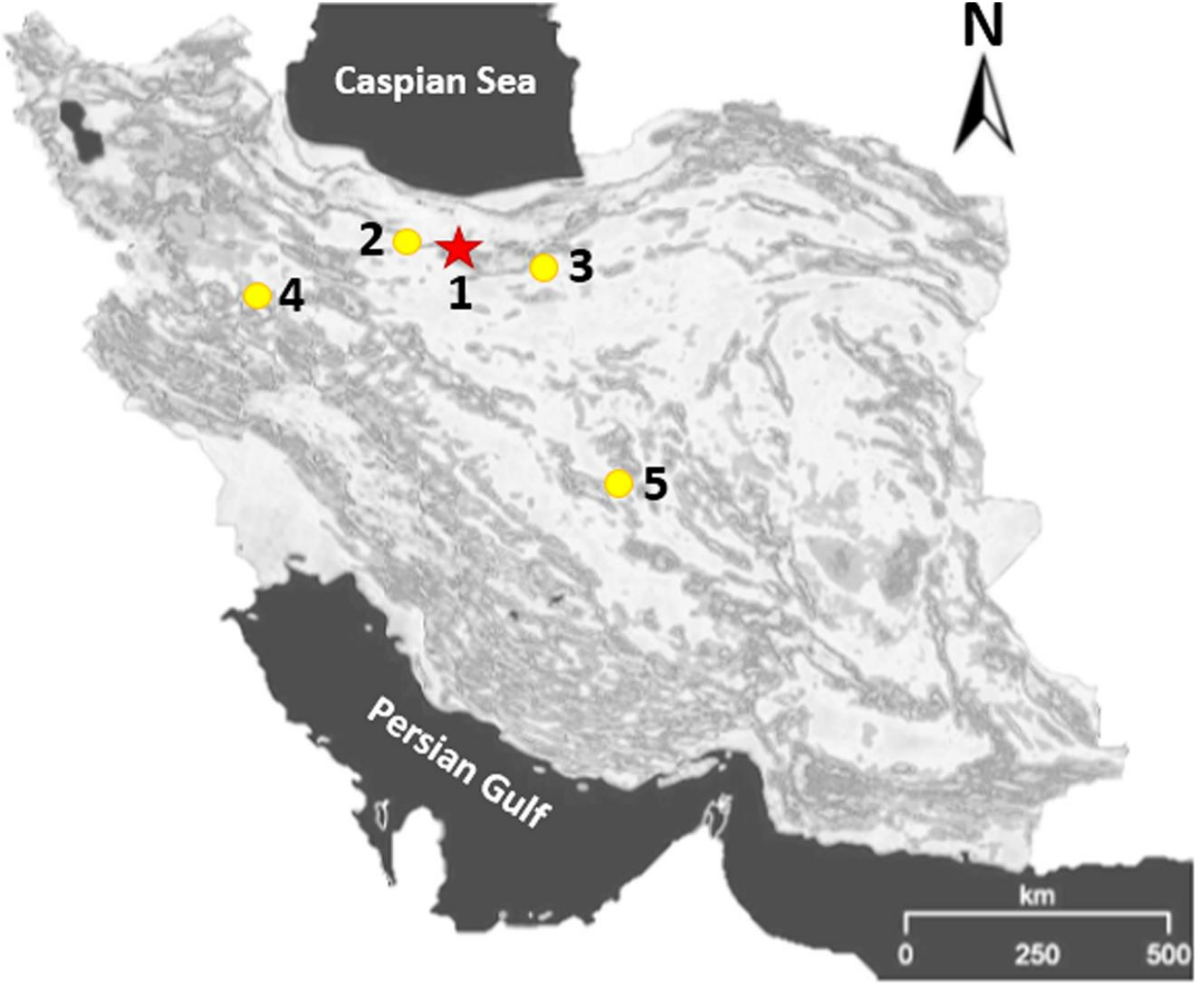
The locations of studied cities inside Iran. Numbers 1, 2, 3, 4, and 5 represent Tehran, Karaj, Semnan, Hamedan, and Yazd, respectively

Bacterial strains were isolated from patients afflicted with gastroenteritis in four different cities (Figure 1) of Iran over the 6-month period of April to September, 2017. Patients could be enrolled if they had had >3 episodes of watery, loose, or bloody stools per day. Details were recorded of the age and gender of each patient from whom fecal samples were retrieved (Table 1). Strains were identified as *S*. Enteritidis using routine biochemical and serological assays (17). Briefly, the stool samples were transferred into Selenite-F broth and incubated at 37 °C for 8 hr. Then, the cultures were again sub-cultured on MacConkey agar. Suspected colonies were subjected to standard biochemical tests including oxidase, catalase, TSI agar, Methyl Red-Voges-Proskauer (MRVP), citrate consumption (Simmons citrate agar), and urease production. Further confirmation of the presumptive *S*. Enteritidis strains was performed by multiplex polymerase chain reaction (PCR) analysis, as described elsewhere (18). Stocks of all strains were stored frozen at -80 °C in cryovials containing TSB complemented with 20% (*v*/*v*) glycerol until required. Bacteria were recovered from freezing by transferring 100 µl into 5 mL of TSB, incubating overnight at 37 °C, and sub-culturing prior to use in assays.

***Antibiotic susceptibility testing***

Kirby-Bauer disk diffusion method was employed in accordance with the guidelines promulgated by the Clinical and Laboratory Standards Institute (19). For this purpose, all of the *S*. Enteritidis strains were screened for resistance to different antibiotics, including amoxicillin (20 μg), cefepime (30 μg), cefotaxime (30 μg), ceftazidime (30 μg), ceftriaxone (30 μg), cefuroxime (30 μg), ciprofloxacin (5 μg), chloramphenicol (30 μg), imipenem (10 μg), meropenem (10 μg), streptomycin (10 μg), tetracycline (30 μg), and trimethoprim/sulfamethoxazole (25 μg). In brief, bacterial suspensions taken from fresh overnight cultures were adjusted to 0.5 McFarland standard and then streaked onto MHA plates. Subsequently, antibiotic disks were placed on each plate. After incubation of MHA plates at 37 °C for 20–24 hr, the zone of growth inhibition surrounding the antibiotic disk was measured. *S*. Enteritidis showing intermediate resistance to antibiotics were considered resistant. MDR was defined as non-susceptibility to at least one antimicrobial agent in three or more antimicrobial categories (20). In all assays,* Escherichia coli* ATCC 25922 was used as quality control in order to ensure the accuracy of susceptibility testing (21).

***Detection of virulence genes***

Total DNA from *S*. Enteritidis strains was extracted by boiling method (22). For this purpose, bacterial strains were harvested from MHA plates, suspended in 200 µl of double-distilled water, and incubated at 100 °C for 15 min. The suspensions were centrifuged at 8000 g for 10 min, after which the supernatant was transferred to another microfuge tube and exploited for subsequent PCR reactions. The primer sequences used for amplification of virulence genes, predicted amplicon sizes, and PCR annealing temperatures of the amplified products are listed in Table 2. Using an Eppendorf thermal cycler (Eppendorf, Germany), extracted DNA (2 μl) was amplified in a 25-μl reaction volume containing 10×PCR buffer (500 mM KCl, 0.8% Nonidet P40, 100 mM Tris–HCl, pH 8.8), 2.5 mM MgCl_2_, 200 μM of deoxynucleotide triphosphates, forward and reverse primers (0.25 μM each), and 1 U of Taq DNA polymerase. After an initial denaturation at 95 °C for 5 min, the cycling conditions were 30 cycles of denaturation at 94 °C for 30 sec, annealing at different temperatures (Table 2) for 30 sec, extension at 72 °C for 1 min, and a 2-min final extension at 72 °C. The PCR products (10 μl) were loaded on a 1.5% (*w*/*v*) ultrapure agarose gel stained with SYBR Safe DNA Gel Stain (Invitrogen, Thermo Fisher Scientific, USA). It was then subjected to electrophoresis in 1×TBE buffer (0.089 M Tris-borate, 0.002 M EDTA; pH 8.0) at 100 V for 40 min. Finally, the gel was visualized with a Gel Doc 2000 gel documentation system (Bio-Rad Laboratories, Hercules, CA, USA).

***DNA sequencing***

One representative of each virulence gene was directly sequenced (Macrogen Research, Seoul, Korea). The corresponding DNA sequences were analyzed online by BLAST software (http://blast.ncbi.nlm.nih.gov/Blast.cgi). Moreover, the DNA sequences were submitted to the EMBL/GenBank databases. *S*. Enteritidis ATCC13076 was used as the control.

***Pulsed-field gel electrophoresis***

All of the *S*. Enteritidis strains were typed by PFGE. Digestion was performed with the restriction enzyme *Xba*I. The resultant restriction fragments were separated with a CHEF-DR III Chiller apparatus (Bio-Rad Laboratories, Hercules, CA, USA) on 1.0% agarose gels (PFGE certified; Bio-Rad) as described previously (33). GelCompare II software version 6.6 (Applied Maths, Austin TX) was also used to examine the patterns. In addition, clustering was performed based upon the unweighted pair group average method (UPGMA) on a Dice coefficient.

## Results

In total, 59 *Salmonella* strains were successfully isolated from 2116 stool samples, which were collected from patients with gastroenteritis in various Iranian cities (including Hamedan, Karaj, Semnan, and Yazd) from April to September 2017. The most prevalent serotype was Enteritidis, accounting for 45.8% (*n* = 27) of all *Salmonella* strains, followed by Paratyphi C (*n* = 12, 20.3%), Typhimurium (*n* = 11, 18.6%), Paratyphi B (*n* = 7, 11.9%), and Paratyphi A (*n* = 2, 3.4%). Out of all 59 patients (*n* = 27), 14 (51.9%) and 13 (48.1%) were males and females, respectively, with ages ranging from 2 to 71 years old (Table 1).

**Figure 2:**
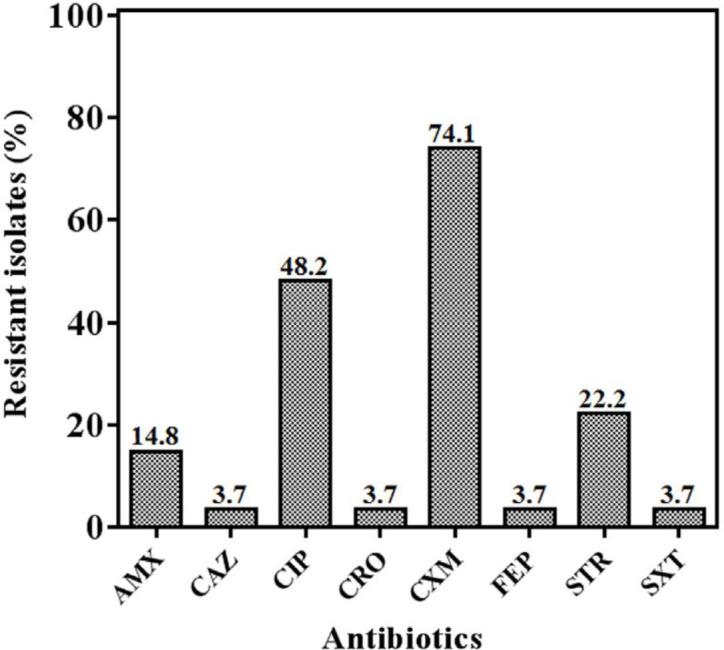
Prevalence of antibiotic resistance in *Salmonella enterica* serotype Enteritidis strains. The bar charts related to cefotaxime, chloramphenicol, imipenem, meropenem, and tetracycline (to which the strains were completely susceptible) are not depicted

AMX: amoxicillin; CAZ: ceftazidime; CIP: ciprofloxacin; CRO: ceftriaxone; CXM: cefuroxime; FEP: cefepime; STR: Streptomycin; TET: tetracycline

Among 27 *S*. Enteritidis strains, 4 MDR strains (14.8%) were detected. We also observed 9 distinct antibiotypes (Table 3), among which Ab4 (simultaneously resistant to ciprofloxacin and cefuroxime) was ranked the most frequent resistance type, followed by Ab8 (only resistant to cefuroxime). Remarkably, almost three-fourths of the strains were resistant to ciprofloxacin (Figure 2). By contrast, resistance towards cefepime, ceftazidime, ceftriaxone, and trimethoprim/sulfamethoxazole was observed less frequently. Cefotaxime, chloramphenicol, imipenem, meropenem, and tetracycline were the antibiotics to which all strains were entirely susceptible. Additionally, 4 strains (14.8%) were found to be sensitive to all 13 tested antibiotics (Table 3).

**Figure 3:**
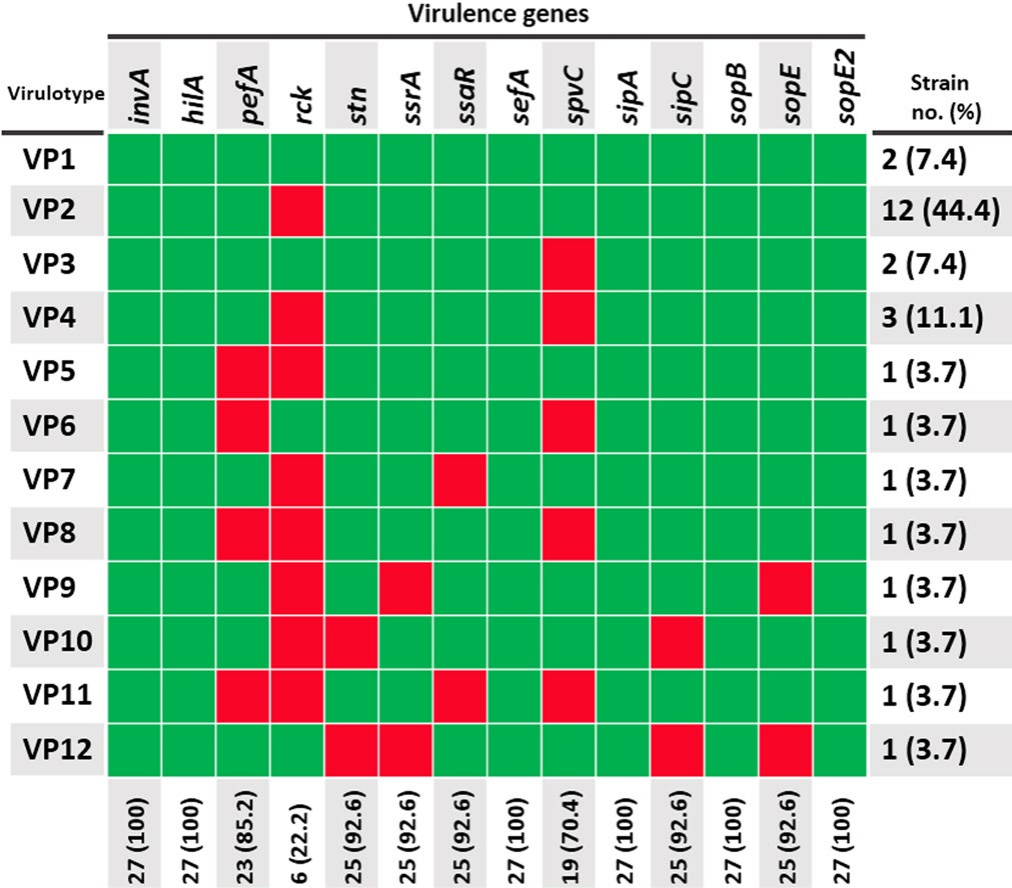
Virulence profiles (virulotypes) of 27 *Salmonella enterica *serotype Enteritidis strains. Green and red squares represent the presence and absence of virulence genes, respectively

With regard to virulence genes, all strains possessed *invA*, *hilA*, *sefA*, *sipA*, *sopB*, and *sopE2* (Figure 3). Other virulence genes including *stn*, *ssrA*, *ssaR*, *sipC*, and *sopE* were each found in 92.6% of strains (*n* = 25). The least prevalent virulence gene was *rck* (*n *= 6; 22.2%). The assigned GenBank accession numbers for virulence genes are KY934465 (*pefA*), KY934468 (*sopB*), KY942079 (*sopE*), KY942080 (*sopE2*), KY942081 (*invA*), KY942082 (*ssaR*), MF004269 (*ssrA*), MF004268 (*hilA*), MF004270 (*stn*), MF033537 (*spvC*), KY934466 (*sipC*), and KY934467 (*rck*).

Based on combinations of virulence genes, 12 virulence profiles (virulotypes) were detected among the strains. As evidenced in Figure 3, VP2 (harboring all genes except for *rck*) was the most common virulotype, occurring in 44.4% of the *S*. Enteritidis strains (*n* = 12).

**Figure 4:**
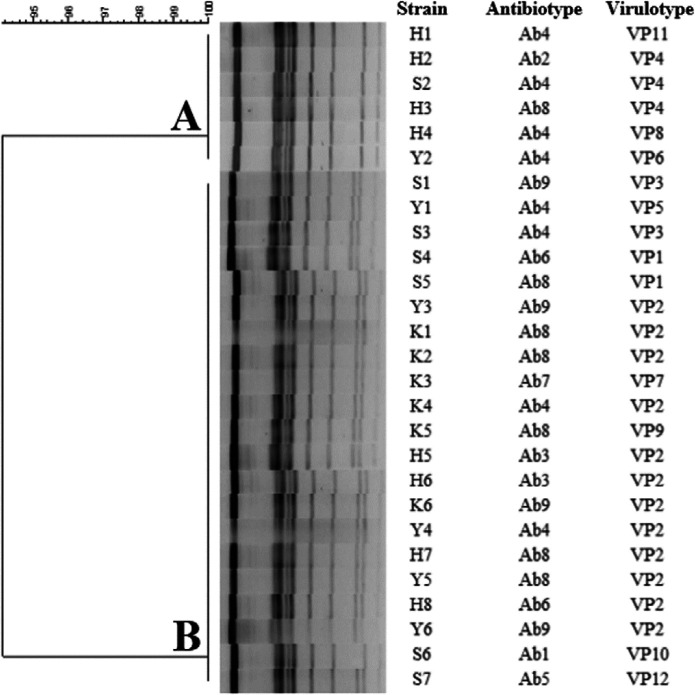
The unweighted pair-group method with arithmetic mean (UPGMA) dendrogram indicates the genetic relatedness among *Salmonella enterica *serotype Enteritidis strains based upon pulsed-field gel electrophoresis fingerprints

PFGE analysis revealed that all of the *S. *Enteritidis strains were divided into two distinct pulsotypes (94% genetic similarity), as illustrated in Figure 4. In this context, the majority of the strains belonged to pulsotype B (*n* = 21), while cluster A contained only 6 strains. The distribution of antibiotypes, virulence profiles, and geographic location of the strains were also compared with that of pulsotypes (Table 4). For instance, pulsotype B comprised strains obtained from all four cities, while no strains from Karaj were observed within pulsotype A. Moreover, all of the Ab8 strains except one shared the same pulsotype. Interestingly, each pulsotype exhibited completely different virulotypes. Of note, VP4 (harboring all genes except* rck *and* spvC*) was only presented in pulsotype A, whereas VP2 was confined to pulsotype B.

**Table 1:** Demographic profiles (age and gender distribution) of the patients together with city and date of isolation for each *Salmonella enterica *serovar Enteritidis strain

*Salmonella enterica *serovar Enteritidis strain			Patients	
ID	City	Date of isolation	Gender	Age
H1	Hamedan	4/14/2017	Female	11
H2	Hamedan	5/10/2017	Male	3
H3	Hamedan	6/5/2017	Male	62
H4	Hamedan	7/23/2017	Female	27
H5	Hamedan	7/26/2017	Male	61
H6	Hamedan	8/31/2017	Male	54
H7	Hamedan	9/5/2017	Female	5
H8	Hamedan	9/17/2017	Male	19
K1	Karaj	5/1/2017	Female	2
K2	Karaj	6/26/2017	Female	71
K3	Karaj	7/8/2017	Female	40
K4	Karaj	8/28/2017	Female	39
K5	Karaj	9/12/2017	Female	64
K6	Karaj	9/23/2017	Female	13
S1	Semnan	4/28/2017	Male	54
S2	Semnan	5/1/2017	Male	9
S3	Semnan	7/13/2017	Female	26
S4	Semnan	8/4/2017	Female	48
S5	Semnan	8/22/2017	Male	67
S6	Semnan	9/15/2017	Male	4
S7	Semnan	9/19/2017	Male	7
Y1	Yazd	7/20/2017	Female	12
Y2	Yazd	7/21/2017	Male	53
Y3	Yazd	8/7/2017	Male	60
Y4	Yazd	8/13/2017	Female	45
Y5	Yazd	8/24/2017	Male	19
Y6	Yazd	9/25/2017	Male	21

**Table 2:** Primers used in the present study for virulence genotyping

Gene	Primer sequences (5ˊ to 3ˊ)	S (bp)	Ann (°C)	Reference
*invA*	F-ACAGTGCTCGTTTACGACCTGAAT R-AGACGACTGGTACTGATCGATAAT	243	60	(23)
*hilA*	F-CGTGAAGGGATTATCGCAGT R-GTCCGGGAATACATCTGAGC	296	56	(24)
*spvC*	F-ACTCCTTGCACAACCAAATGCGGA R-TGTCTCTGCATTTCGCCACCATCA	571	56	(23)
*sipA*	F-CCATTCGACTAACAGCAGCA R-CGGTCGTACCGGCTTTATTA	449	56	(24)
*sopE*	F-CGAGTAAAGACCCCGCATAC R-GAGTCGGCATAGCACACTCA	362	58	(25)
*stn*	F-TTGTCTCGCTATCACTGGCAACC R-ATTCGTAACCCGCTCTCGTCC	617	59	(26)
*pefA*	F-TTGCACTGGGTGGTTCTGG R-TGTAACCCACTGCGAAAG	485	56	(27)
*rck*	F-AACGGACGGAACACAGAGTC R-TGTCCTGACGAAAGTGCATC	189	59	(25)
*sipC*	F-AGACAGCTTCGCAATCCGTT R-ATTCATCCCTTCGCGCATCA	446	61	(24)
*ssaR*	F-GTTCGGATTTGCTTCGG R-TCTCCAGTGACTAACCCTAACCAA	1628	59	(28)
*ssrA*	F-CTTACGATTACGCCATTTACGG R-ATTTGGTGGAGCTGGCGGGAGT	706	58	(29)
*sopB*	F-CCTCAAGACTCAAGATG R-TACGCAGGAGTAAATCGGTG	1987	56	(30)
*sefA*	F-GCAGCGGTTACTATTGCAGC R-TGTGACAGGGACATTTAGCG	321	55	(31)
*sopE2*	F-TCAGGTGGAGCTGTGGA R-TCCAAAAACAGGAAACCACAC	642	56	(32)

S: Size of PCR amplicons; Ann: Annealing temperature

**Table 3:** Antimicrobial resistance profiles among 27 *Salmonella enterica *serotype Enteritidis strains

Antibiotype	Antimicrobial resistance pattern	*n* (%)
Ab1	CIP-CXM-STR-AMX-FEP-CAZ-CRO	1 (3.7)
Ab2	CIP-CXM-STR-TET	1 (3.7)
Ab3	CIP-CXM-STR	2 (7.4)
Ab4	CIP-CXM	8 (29.6)
Ab5	CXM-AMX	1 (3.7)
Ab6	STR-AMX	2 (7.4)
Ab7	CIP	1 (3.7)
Ab8	CXM	7 (25.9)
Ab9	Sensitive to all tested antibiotics	4 (14.8)

AMX: amoxicillin; CAZ: ceftazidime; CIP: ciprofloxacin; CRO: ceftriaxone; CXM: cefuroxime; FEP: cefepime; STR: streptomycin; TET: tetracycline

**Table 4:** Distribution of different antibiotypes and virulotypes among *Salmonella enterica* serotype Enteritidis pulsotypes

Pulsotype	City (*n*)	Antibiotype (*n*)	Virulotype (*n*)
A	Hamedan (4), Karaj (0), Semnan (1), and Yazd (1)	Ab2 (1), Ab4 (4), and Ab8 (1)	VP4 (3), VP6 (1), VP8 (1), and VP 11 (1)
B	Hamedan (4), Karaj (6), Semnan (6), and Yazd (5)	Ab1 (1), Ab3 (2), Ab4 (4), Ab5 (1), Ab6 (2), Ab7 (1), Ab8 (6), and Ab9 (4)	VP1 (2), VP2 (12), VP3 (2), VP5 (1), VP7 (1), VP9 (1), VP10 (1), and VP12 (1)

## Discussion

As a neglected pathogen, *Salmonella* still remains one of the most frequent causative agents of foodborne illnesses throughout the world, representing a substantial economic burden in both developing and high-income countries. According to the Global Burden of Disease Study, an estimated 95 million cases of *Salmonella* enterocolitis occurred in 2017, resulting in more than 50,000 deaths (34). NTS serovars, especially *S*. Typhimurium and *S*. Enteritidis, are responsible for the majority of human salmonellosis cases in Asian, African, European, and North American countries (35). Contaminated foods of animal origin are primary transmission vehicles, though other routes, including person-to-person contact, animal contact, and even environmental exposure may account for a minor fraction of salmonellosis (36). Notwithstanding several epidemiological studies on the prevalence of *S*. Enteritidis in various food samples in Iran (33,37,38), insofar as we know, there is a paucity of published data on the occurrence, antibiotic resistance patterns, and virulence gene profiles of clinical strains in different regions of our country.

In the course of the past decades, antimicrobial resistance has emerged as an eminent global public health issue (8). This study revealed that 14.8% of *S*. Enteritidis strains were resistant to three or more antibiotics. These strains belonged to Ab1 (*n* = 1, 3.7%), Ab2 (*n* = 1, 3.7%), and Ab3 (*n* = 2, 7.4%). The predominant antibiotype was Ab4 (simultaneously resistant to ciprofloxacin and cefuroxime), which accounted for 29.6% of the strains. Cephalosporins and fluoroquinolones are among the therapeutic regimens recommended by clinicians against salmonellosis in humans. Owing to its broad-spectrum activity, fluoroquinolones have become a mainstay for treating severe *Salmonella* infections (39). In the current survey, a marked resistance (48.2%) was noted against ciprofloxacin, which is higher than the rates reported in several studies from Saudi Arabia (40), Greece (41), China (42), the USA (43), and Italy (25). A previous study conducted in Tehran, Iran, reported a nearly negligible resistance rate towards ciprofloxacin (13). However, a later investigation from the same city demonstrated a steep rise in the occurrence of *S*. Enteritidis strains that were not sensitive to ciprofloxacin (14). These results may be attributable to the extensive utilization of quinolones in animal husbandry and farm poultry, resulting in the development and dissemination of quinolone-resistant *Salmonella* strains (44). Consistent with our findings, a high frequency of resistance against cefuroxime (a second-generation cephalosporin) was observed in some recent studies (14, 40, 45). In the current study, a large proportion of *S*. Enteritidis strains still remains susceptible to third- and fourth-generation cephalosporins (i.e., ceftazidime, cefotaxime, ceftriaxone, and cefepime), which corroborates the findings of several investigations from other countries including Saudi Arabia (40), Greece (41), and Brazil (46). However, two previous works from China (42) and Iran (14) reported higher rates of resistance against the newer generations of cephalosporins.

Aminoglycosides have been usually used as growth promoters in animals, though these agents are not recommended for treating salmonellosis (47). In the present work, 22.2% of *S*. Enteritidis strains were resistant to streptomycin. Contrary to our findings, other surveys showed a much greater occurrence of aminoglycoside resistance (13, 14, 40, 42). Nevertheless, two investigations from Brazil (46) and the USA (43) demonstrated very low rates of streptomycin resistance among clinical *S*. Enteritidis. We also found that all of the strains except one were susceptible to trimethoprim-sulfamethoxazole, which is higher than those reported by other studies (13, 14, 40).

Even though carbapenem resistance is being increasingly reported in *Enterobacteriaceae*, it is still extremely rare in *Salmonella* (48). Carbapenems are generally administered as a last resort for treating MDR Gram-negative bacterial infections (48). Fortunately, all the strains were susceptible to imipenem and meropenem in the present study, indicating that these drugs are potentially effective for the treatment of complicated and severe cases of salmonellosis.

The existence of virulence genes has been demonstrated to be robustly associated with *Salmonella* pathogenicity (5). In the present study, all strains (100%) were positive for *invA*, *hilA*, *sefA*, *sipA*, *sopB*, and *sopE2*, indicating that these genes are widespread among clinical *S*. Enteritidis strains. Similar to our findings, previous studies confirmed the presence of *invA* in all strains of *S*. Enteritidis (18, 43, 46, 49, 50). The InvA protein is essential for gut epithelial invasion and its gene sequence is specific to the genus *Salmonella*. HilA, an OmpR/ToxR family transcriptional regulator, directly activates the expression of *invF* and *sicA* (two SPI-1 genes) which encode SPI-1 T3SS apparatus components (10). It is worthwhile to note that *hilA* is highly prevalent among *S*. Enteritidis retrieved from human and food sources (14, 33, 43). As an actin-binding protein, *Salmonella* invasion protein A (SipA) augments the efficiency of the entry process of the pathogen into host cells by influencing the formation of membrane ruffles and rearrangement of the actin cytoskeleton (10). SipB, SipC, and SipD are translocator proteins that are inserted into host membranes and participate in formation of the SPI-1 T3SS needle complex (10,51). SipC also targets F-actin, which is essential for pathogen internalization and invasion (10). *Salmonella* outer proteins (Sops) including SopA, SopB, SopD, SopD2, SopE, and SopE2 are effector proteins that are involved in rearrangement of the cytoskeleton as well as playing a crucial role in inducing inflammation and diarrhea (10). Like other SPI-1 genes, the majority of *S*. Enteritidis strains have been shown to possess genes encoding Sip and Sop proteins (14,46,50).

Interestingly, we found that three plasmid-borne genes (i.e., *pefA*, *spvC*, and *rck*) had the lowest prevalence in comparison with other virulence genes. The plasmid-encoded fimbriae gene (*pefA*) product facilitates bacterial attachment to host epithelial cells, whereas SpvC, a phosphothreonine lyase, is an effector protein that is involved in immune evasion in the early stages of infection as well as dissemination of the pathogen at the later stages (52). *Salmonella* also invades host cells by a Zipper process mediated by Rck, an outer membrane protein that is involved in the invasion process (53). It is generally believed that virulence plasmids are required for severe gastroenteritis and systemic infections (25). Since all of our clinical strains were obtained from stool samples and not from blood or organs, the low frequency of the above-mentioned genes is justified. On the other hand, the majority of our strains (92.6%) carried the *stn* gene, which encodes a heat-labile enterotoxin. The presence of the aforementioned gene in *Salmonella* strains is strongly linked with acute gastroenteritis (54). Previous studies showed that the *stn* gene is widely distributed in various *Salmonella* serovars isolated from diverse sources such as humans, beef, and poultry (14, 26, 33, 50, 55).

Thus far, several genotyping methods such as multilocus enzyme electrophoresis, multilocus sequence typing, multiple-locus variable number of tandem repeat analysis, ribotyping, single nucleotide polymorphism-based approaches, and whole-genome sequencing have been proposed as alternatives to PFGE for outbreaks and source tracking investigations (56). Nevertheless, PFGE is still considered the gold standard for *Salmonella* typing owing to its discriminatory power, reproducibility, ease of execution, and data interpretation (56). In the present study, PFGE analysis revealed that the majority of *S*. Enteritidis strains showed considerable overlap, which is somewhat consistent with the findings of some previous works from Iran (14, 33, 57). Similarly, data from other countries highlighted the lack of genetic diversity in *S*. Enteritidis, suggesting that this serotype is highly clonal (58-60). Despite its usefulness, PFGE usually requires being complemented by other typing methods. In other words, the best discrimination could be achieved for these genetically homogeneous pathogens by using a combination of several typing methods.

## Conclusion

In summary, this study provides insight into antibiotic resistance patterns, virulence factors, and genetic relatedness of clinical *S*. Enteritidis isolated from different cities of Iran. The alarmingly high rates of resistance towards ciprofloxacin and cefuroxime necessitate continuous surveillance of local antibiotic resistance among *Salmonella* strains. The results also showed that the mentioned strains were stemmed from a limited number of clones that had undergone minor genetic changes over time. Future works based upon genetic information, especially whole-genome sequencing, will expand our understanding of *S*. Enteritidis population diversity.
